# Insidious coronary artery disease in a young patient with polyarteritis nodosa: a case report and literature review

**DOI:** 10.1186/s12872-021-01923-9

**Published:** 2021-02-27

**Authors:** Hong Huang, Yanjun Gong, Li Guo, Zhuoli Zhang

**Affiliations:** 1 Department of Rheumatology and Clinical Immunology, Peking University First Hospital, No. 8, Xishiku Street, West District, Beijing, 100034 China; 2 Department of Cardiology, Peking University First Hospital, Beijing, China; 3 Department of Radiology, Peking University First Hospital, Beijing, China

**Keywords:** Polyarteritis nodosa, Coronary heart disease

## Abstract

**Background:**

Polyarteritis nodosa (PAN) is a relatively rare systemic necrotizing vasculitis that typically affects medium-sized arteries. Although myocardial ischemia may occur due to involvement of the coronary arteries, overt myocardial infarction is uncommon.

Case presentation

A 22-year-old Chinese man experiencing chest pain for 7 months was admitted to our hospital. Consistently, the pain tended to last for a few minutes and then spontaneously subside. He had 7-year history of “stable” PAN. Coronary angiography revealed slight plaque infiltration of the left main coronary artery; however occlusion of all the three major coronary arteries with multiple aneurysms. A stent was implanted into the obtuse margin branch artery which was 95% stenosis, and then the chest pain was alleviated. Considering that the occlusion of coronary arteries was due to insidious vasculitis, prednisone 50 mg/day and methotrexate 15 mg/week were reinitiated, in combination with anti-angina medications.

**Conclusions:**

We report a young patient with insidious occlusion of three main coronary arteries under the circumstance of stable PAN for 7 years, suggesting the necessity of assessing the heart, in spite of normal acute phase reactants. The appropriate screening strategy needs to be studied.

## Background

Polyarteritis nodosa (PAN) is a systemic necrotizing vasculitis that typically affects medium-sized arteries, and occasionally small muscular arteries [1]. Involvement of coronary arteries may lead to narrowing or occlusion of arteries, and consequently myocardial ischemia; nevertheless, overt myocardial infarction has been rarely reported [2]. Here, we report a case with 7-year’s history of stable PAN; however insidious occlusion of three main coronary arteries. All relevant literature was also reviewed.

## Case presentation

A 22-year-old Chinese man suffering from chest pain for 7 months with aggravation for 1 month was admitted to our hospital. The pain was intermittent, usually lasted for a few minutes and then subsided spontaneously. He denied any family history of autoimmune diseases and cardiovascular diseases. He has never smoked.

**Fig. 1:**
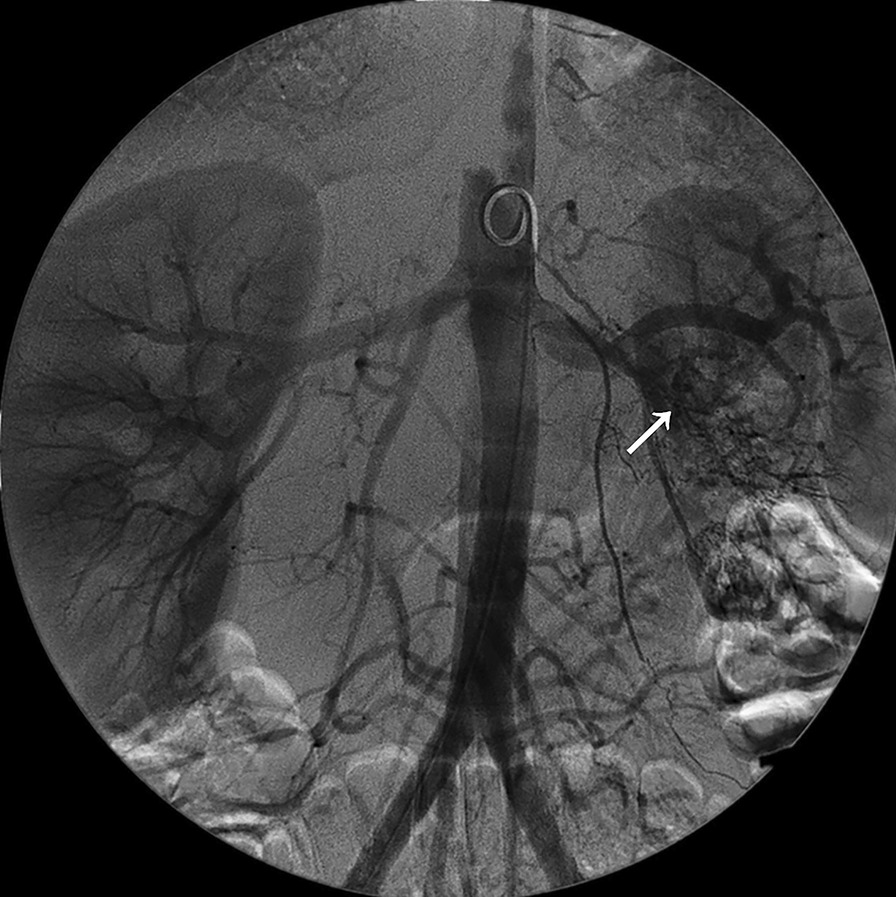
Angiography showed stenosis, occlusion and aneurysmal dilatation of the left renal artery branches (arrow)

Seven years ago, he was diagnosed as PAN due to headache, blurred vision, skin livedo reticularis as well as multiple stenoses, occlusion and aneurysmal dilatation of renal arteries on angiography (Fig. 1). Blood tests showed elevated erythrocyte sedimentation rate (ESR) and C-reactive protein (CRP), but all autoantibodies were negative. He was treated with prednisone in combination with cyclophosphamide, and then rapidly improved. Prednisone was tapered and cyclophosphamide was replaced by azathioprine for maintenance. Since then, he had been in stable condition during regular follow-up of every 6 months. He never discontinued azathioprine during the whole follow-up of 7 years.

**Fig. 2:**
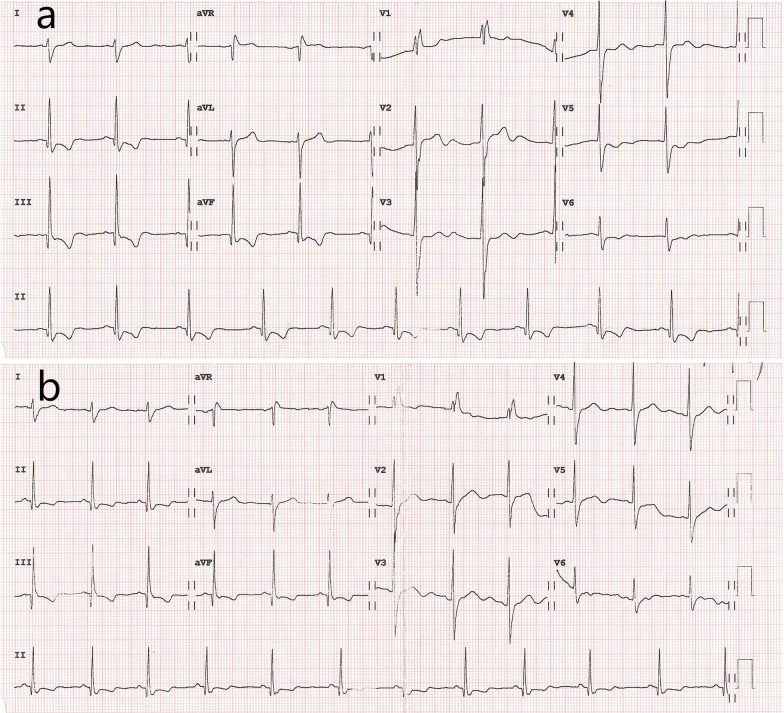
Electrocardiogram before (**a**) and after (**b**) coronary angiography

**Fig. 3:**
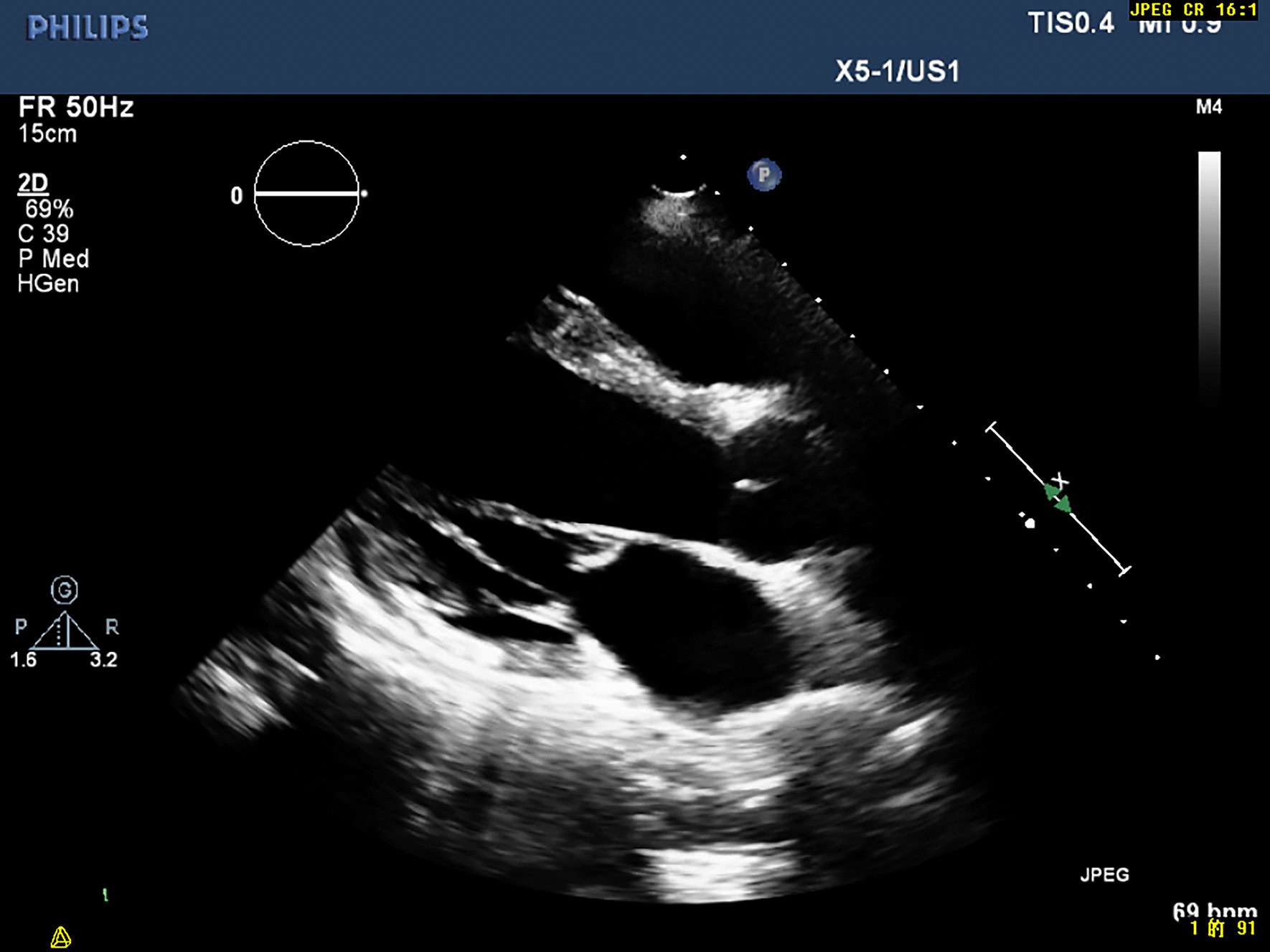
The parasternal long axis views of the left ventricle before coronary angiography

**Fig. 4:**
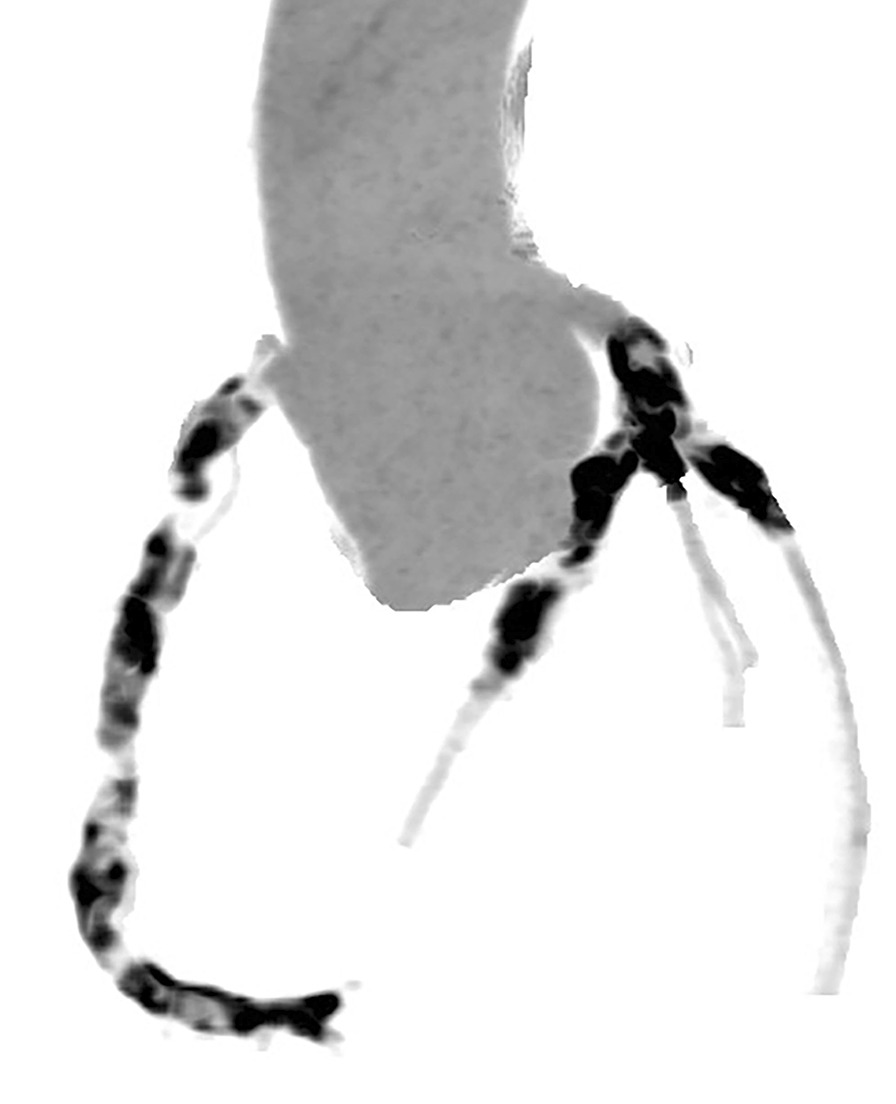
Coronary computed tomography angiography found diffuse coronary stenosis

**Fig. 5:**
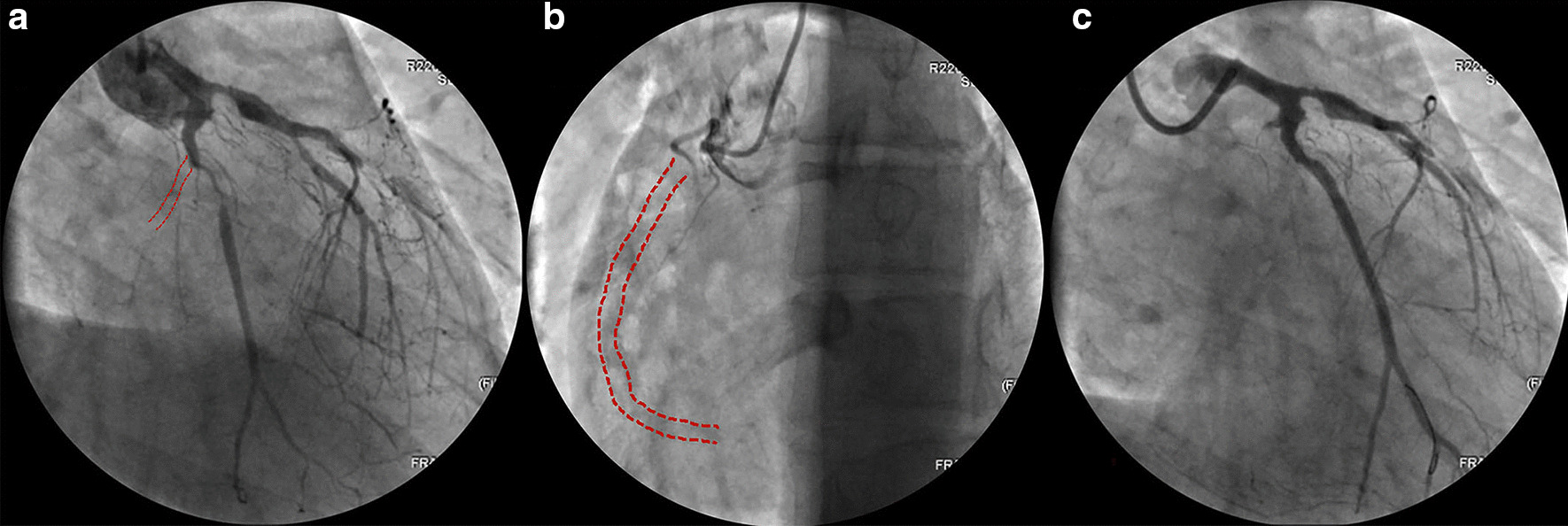
Coronary angiography. **a** 50% stenosis followed by aneurysmal change of the proximal end of left anterior descending (LAD) artery, and complete occlusion of the middle segment; A aneurysmal change of the initial part of left circumflex artery (LCX) and complete occlusion (dotted line); 95% stenosis of obtuse margin branch. **b** totally occluded right coronary artery (dotted line). **c** Appearance of the LCX after stent implantation

**Fig. 6:**
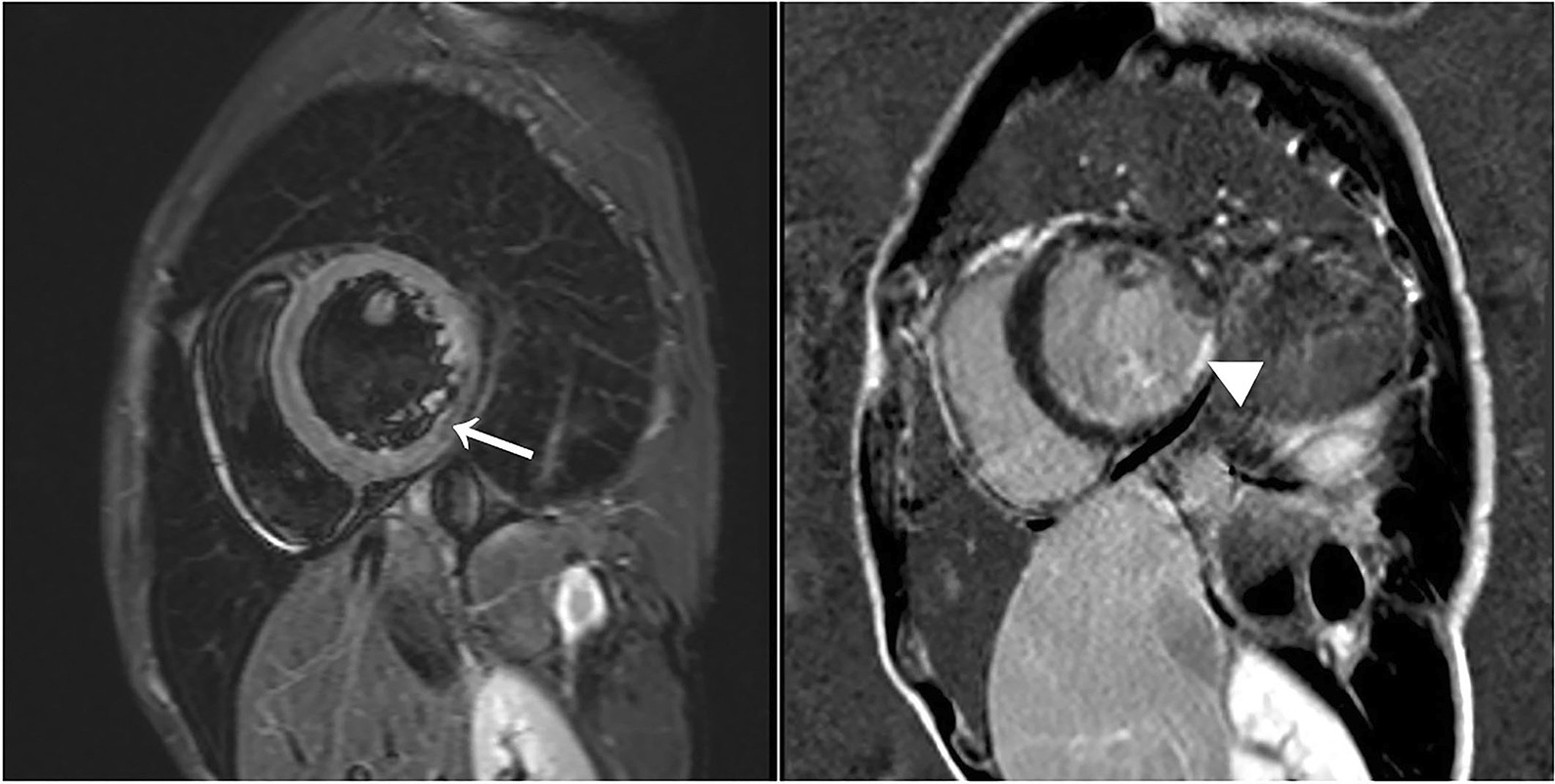
Hypointense on dark blood T2-weighted image (arrow) and late gadolinium enhancement (arrow head) on cardiac magnetic resonance imaging

In December 2019, he suffered from chest distress accompanied by intermittent retrosternal pain, with frequency of approximate 2–3 times a week. The symptoms were gradually worsened with dyspnea sometimes at night. Serological cardiac troponin, ESR and CRP were normal. Electrocardiogram showed right bundle branch block, abnormal Q waves in II, III and avF leads, ST-segment depression and inversion of T waves in II, III, avF and V5–V6 leads (Fig. 2a). Echocardiogram revealed increased left ventricular end-diastolic diameter, as well as echo enhancement and motion abnormality of the inferior posterior wall of the left ventricle (Fig. 3, Additional file 1: Video 1). Coronary computed tomography showed diffused coronary stenosis (Fig. 4). Further coronary angiography revealed slight plaque infiltration of the left main coronary artery, and occlusion of all the three major coronary arteries, with multiple aneurysms. 95% stenosis of the obtuse margin branch artery was also found and a stent was then implanted (Fig. 5). After that, he was getting better and ST-segment depression in III, avF and V5–V6 leads was back to normal (Fig. 2B). Further cardiac magnetic resonance imaging confirmed 25–50% of transmural extent of myocardial infarction with left ventricular ejection fraction of 48% (Fig. 6). Considering that occlusion of coronary arteries was due to insidious vasculitis, prednisone 50 mg daily and methotrexate 15 mg/week were initiated besides anti-angina medications including aspirin and statin. At the recent visit of 9 months, prednisone was tapered to 10 mg/day, while all the other drugs were maintained. He had chest distress occasionally without any retrosternal pain. Serological cardiac troponin, ESR and CRP were normal. Echocardiography showed no obvious deterioration of cardiac structure and function (Additional file 2: Video 2).

## Discussion and conclusions

PAN as a systemic necrotizing vasculitis was first described by Kussmaul and Maier in 1866. Although over a century has passed since its first recognition, heart involvement of PAN is rarely reported. Here we described a young patient with stable PAN for 7 years; however, experiencing chest pain due to coronary occlusion.

All the English literature reporting cardiac involvement in adults with PAN from 1990 to 2019 was reviewed (Additional file 3: Tables 1 and 2). A total of 34 patients were identified from 32 publications. Their average age was 41.2 years (18–74) with a male predominance (24 patients).

There were only 9 (26.5%) patients with a history of PAN when they were experiencing heart problems. PAN was diagnosed after the onset of heart disease for the rest 25 patients, confirmed by imaging or biopsy (11 patients), and by autopsy (14 patients).

A total of 25 (73.5%) patients were admitted to a hospital due to acute coronary syndromes manifesting as chest pain or dyspnea, and 4 (11.8%) patients suffered from abdominal pain or nausea. Among the 12 (35.3%) patients who experienced cardiopulmonary arrest, only 1 patient survived. Nonspecific manifestations such as fever, arthralgia or muscle pain were present in 4 patients (Table 1). The duration of cardiac manifestations from onset to the diagnosis of coronary disease ranged from a few minutes to 1 year.

**Table 1:** Summary of clinical manifestations of the patients reported in the literature

Clinal manifestations	Number
Chest pain or tightness, or dyspnea	25
Abdominal pain or nausea	4
Cardiopulmonary arrest	11
Joint or muscle pain	2
Fever	2

The following abnormalities were found in varied frequencies among the patients with available data, myocardial ischemia on electrocardiogram (15/17 patients), elevated myocardial enzyme (15/17), elevated ESR or CRP (8/12). Coronary arteries were assessed by coronary angiography in 23 patients and autopsy in 14 patients. Stenosis or occlusion of coronary arteries was found in 27 (79.4%) patients, with involvement of single vessel in 11 patients, two vessels in 9 patients, and triple vessels in 7 patients. Aneurysm was found in 12 (35.3%) patients, among whom multiple aneurysms were found in 8 patients. Spontaneous coronary artery dissection was only found in 2 PAN patients. Unexpectely, no abnormality of coronary arteries was found respectively in 1 patient with acute myocardial infarction on angiography or autopsy (Table 2).

**Table 2:** Summary of types of coronary lesions revealed by imaging examinations or autopsy reported in the literature

Types of coronary lesions	Number
Stenosis or occlusion	
Single lesions	
LM	2
LAD	4
RCA	5
Multiple lesions	16
Aneurysms	
Single lesions	4
Multiple lesions	8
Spontaneous coronary artery dissection	2
Normal	2

LM, left main coronary artery; LAD, left anterior descending branch; RCA, right coronary artery

Five patients had been taking glucocorticoid, and/or immunosuppressant when cardiac manifestations occurred, just as our patient. After cardiac involvement was confirmed, most patients received glucocorticoid, and/or immunosuppressant therapy, such as cyclophosphamide or azathioprine, with or without invasive intervention (Table 3). Among the 30 patients with prognosis data, half of them passed away during an average follow-up of 8 months. A total of 11 patients died from cardiac arrest, and 4 patients died from pulmonary edema with alveolar hemorrhage, multiple intracranial hemorrhages after thrombolytic therapy, cardiogenic shock, and acute heart failure, respectively. The relatively common cardiac manifestations of PAN are congestive heart failure, hypertension, pericarditis and arrhythmias [2]. Severe coronary artery involvement—for instance, aneurysm or myocardial infarction, though lethal, has been rarely reported [3, 4]. An autopsy study revealed inflammation of the main coronary arteries and their proximal branches in 62% (41/66) of the patients, but acute myocardial infarction was clinically diagnosed by ante mortem in only 5% of them [4]. Additionally, cardiac symptoms may be the initial manifestations of PAN [5–10]. Arteritis may cause aneurysms and fibrous stenosis of the lumen, extending several centimeters along the coronary artery, which may mimic atherosclerosis [11]. The right coronary artery was most vulnerable, but involvement of other arteries, even multiple artery involvement was also reported [12–16]. Spontaneous coronary artery dissection was uncommon and under-recognized in PAN patients, but should be considered as a differential diagnosis in a patient with acute coronary syndrome or sudden death [3, 17].

**Table 3:** Summary of treatments of the patients reported in the literature

Treatment	Number
Drugs	12
Glucocorticoid	10
Cyclophosphamide	7
Azathioprine	1
Other immunosuppressive agents	1
Surgical operations	6
Stent implantations	4
Endovascular coil treatment	1
No treatment	1

There were also 2 reported PAN cases experiencing acute myocardial infarction with no abnormality found in coronary arteries [18, 19]. Spasm of coronary arteries was probably due to the presence of vasospastic substance in circulation in PAN patients, especially those with Raynaud’s phenomenon [20].

It is noteworthy that the clinical course of coronary involvement in most reported PAN cases was very short, while imaging or autopsy often indicated presence of chronic lesions, suggesting the insidious nature of coronary artery involvement in PAN. Moreover, the coronary artery lesions sometimes occurred when systemic inflammation of vasculitis appeared to be inactive. This was the case of our patient as well as other reported cases [2, 12, 14, 21]. Travers found that there was no correlation between clinical variables and presence of aneurysm in a given organ/system [22]. Clinical symptoms and angiographic changes seem to be unparallel.

As there were few cases on coronary involvement in the context of PAN, the pathogenesis and optimal treatment strategies of PAN-related lesions were insufficiently studied. Immunosuppression treatment is fundamental to control inflammation. Unfortunately, coronary artery involvement can still happen after PAN has been stable with Immunosuppression treatment. Yamamoto et al. also reported a case with triple coronary artery involvement when PAN was stable [12]. How to judge disease activity as well as monitor therapeutic response is currently challengeable. Coronary artery bypass grafting (CABG) using a saphenous vein graft was ever tried without any medications. CABG using left internal mammary artery together with saphenous vein graft was also reported in 2 PAN cases [13, 21]. Although the short-term efficacy of these interventions was good, the long-term outcome of CABG was unknown.

In conclusion, we report a young PAN patient with insidious occlusion of three main coronary arteries under the circumstance of stable PAN for 7 years. This reminds us of the necessity of assessing vessels in PAN patients, in spite of normal acute phase reactants. The appropriate screening strategy needs to be studied.

## Supplementary Information

**Additional file 1.** **Table 1.** Manifestations of patients with polyarteritis nodosa in published reports.  **Table 2.** Treatments and outcomes of the patients in published reports.

**Additional file 2.** **Supplementary video 1.** Echocardiography before stent implantation.

**Additional file 3.** **Supplementary video 2.** Echocardiography after stent implantation.

## Data Availability

Data are available from Zhuoli Zhang (E-mail: zhuoli.zhang@126.com) upon reasonable request and with permission of Peking University First Hospital.

## Abbreviations

PAN: Polyarteritis nodosa
ESR: Erythrocyte sedimentation rate
CRP: C-reactive protein
CABG: Coronary artery bypass grafting
