# Supplementary material for: Insidious coronary artery disease in a young patient with polyarteritis nodosa: a case report and literature review
Source: BMC Cardiovasc Disord. 2021 Feb 27;21:115. doi: 10.1186/s12872-021-01923-9 (PMC7912834; doi:10.1186/s12872-021-01923-9)
Supplement: Supplementary file 3 — Additional file 3. Supplementary video 2. Echocardiography after stent implantation. [file 12872_2021_1923_MOESM3_ESM.docx]

**Supplementary File**

Additional Supporting Information may be found in the online version of this article at the publisher’s website.

**SUPPLEMENTARY APPENDIX**

**Table 1.** Manifestations of patients with polyarteritis nodosa in published reports

**Table 2.** Treatments and outcomes of the patients in published reports

Table 1. Manifestations of patients with polyarteritis nodosa in published reports

| No | Author | Age(yrs)/  Gender | When heart disease occurred | |  | Manifestations | | | | | | |
| --- | --- | --- | --- | --- | --- | --- | --- | --- | --- | --- | --- | --- |
|  |  |  | PAN course | Treatments |  | Symptoms | Course | ECG findings | Myocardial enzymes | Acute-phase proteins | Imaging findings | Autopsy findings |
| 1 | Sakai K [1] | 34/M | not diagnosed | no treatment |  | severe nausea, cardiopulmonary arrest | sudden onset | NA | NA | NA | NA | thrombotic occlusion in the LM and LAD, and mild stenosis (50% in diameter) in the LCX and RCA |
| 2 | Gabarrou G [2] | 52/M | not diagnosed | no treatment |  | chest pain, cardiopulmonary arrest | a few minutes | NA | NA | NA | NA | tight stenosis of the anterior interventricular artery (greater than 90%), atherosclerosis in all coronary artery walls, the diameter of all coronary artery walls was reduced but stenosis was not greater than 50% |
| 3 | Lewandowski M [3] | 29/M | 17 years | low-dose glucocorticoids |  | ACS | 4 months | NA | NA | NA | stenoses within LAD, RCA and LCX | - |
| 4 | Indaram MB [4] | 38/F | NA | immunosuppressive therapy |  | intermittent angina pectoris | NA | NA | NA | NA | multiple large, partially thrombosed aneurysms within the major epicardial vessels | - |
| 5 | Yamamoto Y [5] | 38/M | 23 years | no treatment |  | exertional chest tightness | 6 months | NA | NA | normal CRP and ESR | an aneurysmal change of the LM, LAD, and LCX; total occlusion of the proximal RCA; 99% stenotic lesions of the LAD and PL | - |
| 6 | Munguti CM [6] | 62/F | not diagnosed | no treatment |  | severe abdominal pain associated with nausea and vomiting, then sudden cardiac arrest | 1 day | NA | NA | NA | NA | The cause of death was suspected to be due to cardiac arrhythmias from PDA occlusion since myocardial sections did not show any evidence of new or old ischemic injury |
| 7 | Bayturan Ö [7] | 36/M | not diagnosed | no treatment |  | stable angina pectoris and claudication of the right lower | NA | NA | NA | NA | a chronic total occlusion of the RCA stent with bridging collaterals; nonobstructive lesions within the LCX; a large CAA in the LAD | - |
| 8 | Reindl M [8] | 49/M | not diagnosed | no treatment |  | abdominal pain | 1 day | sinus rhythm with new repolarization abnormalities (inverted T-waves) in aVF and III | elevated high-sensitivity cardiac troponin T | elevated CRP (196 mg/l) | a complete thrombotic occlusion of the RCA | - |
| 9 | Ebersberger U [9] | 35/F | NA | NA |  | recurrent chest pain | NA | NA | NA | NA | a vast aneurysm of RCA | - |
| 10 | Kawajiri H [10] | 46/F | 12 years | no treatment |  | cardiopulmonary arrest | sudden onset | ventricular fibrillation, then pulseless electrical activity, after first aid it showed ST segment elevation in leads V1 to V5 | NA | NA | occlusion of the proximal RCA and LAD, as well as multiple CAAs | - |
| 11 | Dermengiu D [11] | 18/M | not diagnosed | no treatment |  | felt fatigued when playing football and immediately collapsed | sudden onset | acute anterolateral myocardial infarction with ST elevation | NA | NA | NA | a segmentary coronary vasculitis which led to vascular thrombosis and myocardium infarction |
| 12 | Harada Y [12] | 71/M | not diagnosed | no treatment |  | chest pain, sustained fever, dry mouth, appetite loss, pain in back, cardiac arrest | 3 months | negative T-waves in the V4, V5 and V6 leads | NA | CRP 24.53 mg/dl | NA | no sclerotic changes, narrowing or occlusion.; RCA, LAD and their branches showed remarkable infiltration of granulocytes |
| 13 | McWilliams ET [13] | 31/M | not diagnosed | no treatment |  | atypical left-sided chest pain | NA | normal | cardiac Troponin I was elevated at 4.57 | elevated CRP and ESR | an abnormality in the mid-LAD suggestive of microaneurysm formation | - |
| 14 | Shields LB [14] | 32/M | not diagnosed | no treatment |  | lower left quadrant abdominal pain, found unresponsive at home, apneic and pulseless | 3 weeks | NA | NA | NA | NA | focal areas of myocardial infarct primarily of the left ventricle |
| 15 | Canpolat U [15] | 23/F | 15 years | cyclophosphamide (50 mg twice daily)， prednisolone (5 mg every other day) |  | retrosternal chest pain | NA | acute inferior MI with no right ventricular infarction | elevated | normal CRP and ESR | coronary ectasia in LAD and LCX, RCA was occluded from the proximal segment | - |
| 16 | Chung DC [16] | 52/M | not diagnosed | no treatment |  | resting chest discomfort | NA | Q wave at lead III | CK 38IU/L (normal), CK-MB 0.7ng/ml (normal) | ESR 76mm/hour, high-sensitivity CRP 94.9mg/L | large aneurysms on LAD and LCX with CTO on distal segments; RCA showed huge aneurismal change with CTO lesion | - |
| 17 | Brooks MJ [17] | 25/F | 3years | prednisolone |  | cardiac arrest due to ventricular fibrillation | sudden onset | marked anterolateral ST-segment depressions | NA | NA | severe generalized coronary arteritis; critical stenoses of the proximal LAD and the posterior left ventricular branch | - |
| 18 | Yuce M [18] | 29/F | not diagnosed | no treatment |  | chest pain and joint pain | NA | ST segment depression in leads V4 to V6 | NA | NA | numerous aneurysms involving both the epicardial and intramyocardial segments of LAD, LCX and RCA, and RCA was occluded | - |
| 19 | Moraes RC [19] | 74/F | not diagnosed | no treatment |  | sudden dyspnea accompanied by lower-limb edema, pain, paresthesia and weakness | 5 days | ST-segment elevation from V1 to V6, I and aVL | CK-MB = 49.7 ng/mL, troponin I = 1.46 ng/mL | NA | 40% stenosis in the RCA, in the diagonal branch and in the first diagonal branch of LM | occluded epicardial coronary branch; acute thrombosis of the distal segments of the anterior interventricular artery and the right anterior ventricular arteries |
| 20 | Yanagawa B [20] | 46/M | 6 years | oral prednisone and cyclophosphamide |  | facial and jaw discomfort with mild associated dyspnea | NA | NA | NA | normal CRP and ESR | a rather large diameter, and mildly aneurysmal; LM with multiple stenoses; the proximal LAD, stented diagonal, proximal LCX, and second OM all demonstrated 90% lesions and the proximal RCA had a 70% lesion | - |
| 21 | Wi J [21] | 31/F | not diagnosed | no treatment |  | intermittent chest pain | NA | ST-segment depressions in precordial leads V1-V6 | a rise in creatine kinase-MB to 121.2 ng/mL and in troponin-T to 1.73 ng/mL | CRP 4mg/dl, ESR 31mm/hour | huge multiple aneurysmal changes involving the LM and all three major coronary arteries；LCX was totally occluded at its ostium; critical stenotic lesions were found at the distal LAD and the posterolateral branch of RCA | - |
| 22 | Marla R [22] | 61/M | not diagnosed | no treatment |  | stable angina | NA | NA | NA | NA | a giant aneurysm in the proximal LAD leading to 2 diagonal branches and total occlusion of the LAD; a giant aneurysm on the LCX beyond its takeoff from LM; the dominant RCA had a 95% proximal stenosis and a giant aneurysm distal to the stenosis | - |
| 23 | Uçar HI [23] | 36/M | 7 years | NA |  | chest pain | NA | acute myocardial ischemic changes | NA | NA | 90% stenosis in proximal LAD and total occlusion of RCA | - |
| 24 | Wagner AD [24] | 49/M | not diagnosed | no treatment |  | symmetrical joint and muscle pain | 4.5 months | ST segment in the anterior leads | elevated CK of 1425IU/l and CK-MB 31IU/l, troponin I was markedly raised | ESR 120mm/hour, CRP 306.3mg/l | a 90% stenosis of LAD | - |
| 25 | Kastner D [25] | 35/M | not diagnosed | prednisone and cyclophosphamide for eczematous dermatitis with peripheral eosinophilia |  | precordial chest pain | NA | acute anterolateral myocardial infarction | elevated CK level of 800U/L | ESR 4mm/hour | a proximal stenosis of 70-80% in RCA and was diffusely diseased with aneurysmal dilations; large proximal aneurysms in LAD and LCX | - |
| 26 | Chu KH [26] | 51/F | not diagnosed | no treatment |  | sudden onset sharp retrosternal chest pain | sudden onset | acute anterolateral myocardial infarction | normal | ESR 38mm/hour | a spontaneous dissection measuring 30mm in the distal LAD | - |
| 27 | Srinivasan G [27] | 49/M | not diagnosed | no treatment |  | band-like chest pain | 1 hour | ST-segment elevation noted in leads I, aVL, V5, and V6 | NA | NA | a high-grade stenosis (90%) of the first diagonal artery, borderline stenosis in a large ramus intermedius artery and with 50% stenosis in its two main branches, and a 50% stenosis in the second diagonal artery | fibrinoid necrosis of LAD |
| 28 | Odhav S [28] | 44/M | not diagnosed | no treatment |  | increasing angina | 2 days | compatible with anterolateral MI | compatible with anterolateral MI | NA | occlusion of LAD | vasculitis of PAN in many organs, including the heart and coronary arteries |
| 29 | Nakazawa K [29] | 73/M | not diagnosed | no treatment |  | intermittent fever, severe intermittent pain appeared in the right upper abdominal quadrant and lower abdomen | 1 year | NA | NA | ESR 62mm/hour, CRP 18.2mg/dl | NA | The coronary arteries showed necrotizing arteritis with narrowing of the lumina and thrombotic occlusion |
| 30 | Rajani RM [30] | 20/M | not diagnosed | no treatment |  | acute excruciating substernal chest pain, breathlessness, poor peripheral circulation | 2 hours | extensive anterior wall myocardial infarction | marked elevation of serum CK-MB fraction | NA | normal coronary arteries | - |
| 31 | Swalwell CI [31] | 47/M | not diagnosed | no treatment |  | chest pains and shortness of breath, cardiopulmonary arrest | NA | NA | NA | NA | NA | extensive concentric narrowing of all three major coronary arteries extending from the level of the ostia |
|  |  | 26/M | not diagnosed | no treatment |  | pain of chest and arms, shortness of breath, cardiopulmonary arrest | sudden onset | NA | NA | NA | NA | a striking concentric narrowing of the LM diffusely between the ostium and bifurcation |
|  |  | 27/M | not diagnosed | no treatment |  | chest pain, cardiopulmonary arrest | 1 evening | NA | NA | NA | NA | 3-cm long acute thrombotic occlusion of LAD |
| 32 | Paul RA [32] | 33/M | not diagnosed | no treatment |  | fatigue and occasional thoracoabdominal pain | 1 year | NA | NA | NA | NA | LM was almost totally occluded |

NA, not available; LM, left main coronary artery; LAD, left anterior descending artery; LCX, left circumflex artery; RCA, right coronary artery; PL, posterior branches of left ventricular; PDA, posterior descending artery; OM, obtuse marginal branch; ACS, acute coronary syndrome; MI, myocardial infarction; CAA, coronary artery aneurysm; CTO, chronic total occlusion; ECG, electrocardiogram; CRP, C-reactive protein; ESR, erythrocyte sedimentation rate; CK, creatinine kinase; MB, muscle-brain isoenzyme.

Table 2. Treatments and outcomes of the patients in published reports

| No | Author | Age(yrs)/  Gender | Treatments | Outcomes | Follow-up time |
| --- | --- | --- | --- | --- | --- |
|  |  |  |  |  |  |
| 1 | Sakai K [1] | 34/M | - | died | - |
| 2 | Gabarrou G [2] | 52/M | - | died | - |
| 3 | Lewandowski M [3] | 29/M | stents were implanted, intensive anti-inflammatory treatment (methylprednisolone and cyclophosphamide) | no angina has been observed | NA |
| 4 | Indaram MB [4] | 38/F | NA | NA | NA |
| 5 | Yamamoto Y [5] | 38/M | CABG using an SVG to the LAD and an SVG to the PL | symptoms disappeared after surgery | NA |
| 6 | Munguti CM [6] | 62/F | - | died | - |
| 7 | Bayturan Ö [7] | 36/M | endovascular coil treatment | angina free | 6 weeks |
| 8 | Reindl M [8] | 49/M | stent implantation; immunosuppressive treatment (methylprednisolone) | an improved clinical condition | a few weeks |
| 9 | Ebersberger U [9] | 35/F | a single CABG using a SVG; intravenous cyclophosphamide | the patient was discharged in good condition to rehabilitation | NA |
| 10 | Kawajiri H [10] | 46/F | CABG using LIMA, no immunosuppressive treatment | the internal mammary artery graft displayed good patency, and the coronary artery aneurysm had not progressed | 14 months |
| 11 | Dermengiu D [11] | 18/M | - | died | - |
| 12 | Harada Y [12] | 71/M | - | died | - |
| 13 | McWilliams ET [13] | 31/M | intravenous methylprednisolone and cyclophosphamide | remained well | NA |
| 14 | Shields LB [14] | 32/M | - | died | - |
| 15 | Canpolat U [15] | 23/F | stent implantation; no additional immune-suppressive therapy | asymptomatic with no ischemia | one year |
| 16 | Chung DC [16] | 52/M | immunosuppressive agents | NA | NA |
| 17 | Brooks MJ [17] | 25/F | azathioprine and prednisolone | died | several months |
| 18 | Yuce M [18] | 29/F | NA | NA | NA |
| 19 | Moraes RC [19] | 74/F | - | Died of pulmonary edema and  alveolar hemorrhage | - |
| 20 | Yanagawa B [20] | 46/M | CABG using LIMA and SVGs | NA | NA |
| 21 | Wi J [21] | 31/F | high-dose steroids (prednisolone 60 mg/d) and an immunosuppressive agent (cyclophosphamide 100 mg/d) | remained asymptomatic without complications | 6 months |
| 22 | Marla R [22] | 61/M | surgical operation | no symptoms anymore | 18 months |
| 23 | Uçar HI [23] | 36/M | coronary artery bypass grafting | uneventful | NA |
| 24 | Wagner AD [24] | 49/M | stent implantation; intravenous methylprednisolone with continuous oral prednisolone; intravenous cyclophosphamide | no relapse | 6 months |
| 25 | Kastner D [25] | 35/M | cyclophosphamide 50mg TID and prednisone 40mg QD | uneventful | NA |
| 26 | Chu KH [26] | 51/F | prednisone 60 mg QD and cyclophosphamide 50 mg BID | in good condition | 32 days |
| 27 | Srinivasan G [27] | 49/M | thrombolytic therapy | died of multiple intracranial hemorrhages after thrombolytic therapy | - |
| 28 | Odhav S [28] | 44/M | intravenous methylprednisolone | died in cardiogenic shock | NA |
| 29 | Nakazawa K [29] | 73/M | prednisone | died of acute heart failure | - |
| 30 | Rajani RM [30] | 20/M | no treatment for PAN | no evidence of ongoing myocardial ischemia | 6 months |
| 31 | Swalwell CI [31] | 47/M | - | died | - |
|  |  | 26/M | - | died | - |
|  |  | 27/M | - | died | - |
| 32 | Paul RA [32] | 33/M | - | died | - |

NA, not available; LAD, left anterior descending artery; PL, posterior branches of left ventricular; CABG, coronary artery bypass grafting; SVG, saphenous vein graft; LIMA, left internal mammary artery.

**Reference**

1. Sakai K, Asakura K, Saito K, Fukunaga T: **Sudden unexpected death due to coronary thrombosis associated with isolated necrotizing vasculitis in the coronary arteries of a young adult**. *Forensic Sci Med Pathol* 2019, **15**(2):252-257.

2. Gabarrou G, Guilbeau-Frugier C, Blanc A, Telmon N, Savall F: **Sudden Death Due to Coronary Arteritis**. *J Forensic Sci* 2018, **63**(2):611-613.

3. Lewandowski M, Goracy J, Kossuth I, Peregud-Pogorzelska M: **Vasculitis or coronary atherosclerosis? Optical coherence tomography images in polyarteritis nodosa**. *Kardiol Pol* 2018, **76**(4):813.

4. Indaram MB, Saeed IM, Patil H, Thompson RC: **The "Nodes" in Polyarteritis Nodosa-Coronary Artery Aneurysms**. *J Clin Rheumatol* 2018.

5. Yamamoto Y, Iino K, Ueda H, No H, Nishida Y, Takago S, Shintani Y, Kato H, Kimura K, Takemura H: **Coronary Artery Bypass Grafting in a Patient With Polyarteritis Nodosa**. *Ann Thorac Surg* 2017, **103**(5):e431-e433.

6. Munguti CM, Ndunda PM, Muutu TM: **Sudden Death from Spontaneous Coronary Artery Dissection due to Polyarteritis Nodosa**. *Cureus* 2017, **9**(10):e1737.

7. Bayturan O, Tarhan S, Copkiran O, Duzgun F, Tezcan UK: **Endovascular coil treatment of a coronary artery aneurysm related to polyarteritis nodosa**. *Anatol J Cardiol* 2017, **18**(5):370-372.

8. Reindl M, Reinstadler SJ, Feistritzer HJ, Mayr A, Klug G, Marschang P, Metzler B: **Acute myocardial infarction as a manifestation of systemic vasculitis**. *Wien Klin Wochenschr* 2016, **128**(21-22):841-843.

9. Ebersberger U, Rieber J, Wellmann P, Goebel C, Gansera B: **Polyarteritis nodosa causing a vast coronary artery aneurysm**. *J Am Coll Cardiol* 2015, **65**(5):e1-2.

10. Kawajiri H, Koh E, Masuda N, Kira H, Yamasaki T: **Coronary artery bypass grafting in a patient with polyarteritis nodosa presenting with acute myocardial infarction and multiple coronary aneurysms**. *Ann Thorac Cardiovasc Surg* 2014, **20 Suppl**:769-772.

11. Dermengiu D, Hostiuc S, Cristian Curca G, Constantin Rusu M, Paparau C, Ceausu M: **Sudden death due to isolated segmentary coronary vasculitis**. *Am J Forensic Med Pathol* 2014, **35**(4):223-231.

12. Harada Y, Suzuki T, Shinagawa T, Yoshimoto T: **Cardiac arrest in a patient with polyarteritis nodosa**. *Intern Med* 2013, **52**(24):2759-2763.

13. McWilliams ET, Khonizy W, Jameel A: **Polyarteritis nodosa presenting as acute myocardial infarction in a young man: importance of invasive angiography**. *Heart* 2013, **99**(16):1219.

14. Shields LB, Burge M, Hunsaker JC, 3rd: **Sudden death due to polyarteritis nodosa**. *Forensic Sci Med Pathol* 2012, **8**(3):290-295.

15. Canpolat U, Dural M, Atalar E: **Acute inferior myocardial infarction in a young female patient with polyarteritis nodosa**. *Herz* 2012, **37**(4):461-463.

16. Chung DC, Choi JE, Song YK, Lim AL, Park KH, Choi YJ: **Polyarteritis nodosa complicated by chronic total occlusion accompanying aneurysms on all coronary arteries**. *Korean Circ J* 2012, **42**(8):568-570.

17. Brooks MJ, Iyer R: **Images in clinical medicine. Coronary arteritis**. *N Engl J Med* 2012, **367**(7):658.

18. Yuce M, Davutoglu V, Sari I, Onat AM: **Polyarteritis nodosa with multiple coronary aneurysms presenting as acute myocardial infarction**. *Am J Med Sci* 2011, **341**(5):409.

19. Moraes RC, Mangili LC, Benvenuti LA: **Case 1 /2011--seventy-four-year-old female patient with sudden apnea and acute cholecystitis, five days after acute myocardial infarction without critical coronary lesions**. *Arq Bras Cardiol* 2011, **96**(3):e35-41.

20. Yanagawa B, Kumar P, Tsuneyoshi H, Kachel E, Massad E, Moussa F, Cohen GN: **Coronary artery bypass in the context of polyarteritis nodosa**. *Ann Thorac Surg* 2010, **89**(2):623-625.

21. Wi J, Choi HH, Lee CJ, Kim T, Shin S, Ko YG, Jang Y, Park YB, Kwon YJ: **Acute Myocardial Infarction due to Polyarteritis Nodosa in a Young Female Patient**. *Korean Circ J* 2010, **40**(4):197-200.

22. Marla R, Ebel R, Crosby M, Almassi GH: **Multiple giant coronary artery aneurysms**. *Tex Heart Inst J* 2009, **36**(3):244-246.

23. Ucar HI, Oc M, Tok M, Ozyuksel A, Oc B, Farsak B: **Coronary artery bypass with saphenous vein graft in a middle-aged patient with polyarteritis nodosa**. *Anadolu Kardiyol Derg* 2007, **7**(2):231-232.

24. Wagner AD, Feist T, Prondzinsky R, Fleig WE, Keysser G: **Joint and muscle pain with mononeuritis multiplex, tetraparesis, and myocardial infarction in a previously healthy adult**. *Ann Rheum Dis* 2001, **60**(11):1003-1006.

25. Kastner D, Gaffney M, Tak T: **Polyarteritis nodosa and myocardial infarction**. *Can J Cardiol* 2000, **16**(4):515-518.

26. Chu KH, Menapace FJ, Blankenship JC, Hausch R, Harrington T: **Polyarteritis nodosa presenting as acute myocardial infarction with coronary dissection**. *Cathet Cardiovasc Diagn* 1998, **44**(3):320-324.

27. Srinivasan G, Boschman C, Roth SI, Hendel RC: **Unsuspected vasculitis and intracranial hemorrhage following thrombolysis**. *Clin Cardiol* 1997, **20**(1):84-86.

28. Odhav S, McKown K, Lohr KM: **Polyarteritis nodosa presenting as recurrent myocardial infarction**. *Chest* 1994, **105**(5):1615.

29. Nakazawa K, Itoh N, Duan HJ, Komiyama Y, Shigematsu H: **Polyarteritis nodosa with atrophy of the left hepatic lobe**. *Acta Pathol Jpn* 1992, **42**(9):662-666.

30. Rajani RM, Dalvi BV, D'Silva SA, Lokhandwala YY, Kale PA: **Acute myocardial infarction with normal coronary arteries in a case of polyarteritis nodosa: possible role of coronary artery spasm**. *Postgrad Med J* 1991, **67**(783):78-80.

31. Swalwell CI, Reddy SK, Rao VJ: **Sudden death due to unsuspected coronary vasculitis**. *Am J Forensic Med Pathol* 1991, **12**(4):306-312.

32. Paul RA, Helle MJ, Tarssanen LT: **Sudden death as sole symptom of coronary arteritis**. *Ann Med* 1990, **22**(3):161-162.
